# Utilization of potato peels waste for production, purification, and characterization of cold-adapted amylase under solid state fermentation by *Penicillium goetzii* AUMC 498

**DOI:** 10.1186/s12934-026-03021-x

**Published:** 2026-05-18

**Authors:** Rabab Shobak Sakr, Ahmed M. Moharram, Heba Atia Yassa, Bahaa E. S. Abd El-Fatah, Osama A. M. Al-Bedak

**Affiliations:** 1 Molecular Biology Research Institute, Assiut University, Assiut, 71511 Egypt; 2 Assiut University Mycological Centre, Assiut University, Assiut, 71511 Egypt; 3 Department of Botany and Microbiology, Faculty of Science, Assiut University, Assiut, 71511 Egypt; 4 Department of Forensic Medicine and Clinical Toxicology, Faculty of Medicine, Assiut University, Assiut, 71511 Egypt; 5 Faculty of Biotechnology, Badr University in Cairo (BUC), Badr City, Cairo 11829 Egypt; 6 Department of Genetics, Faculty of Agriculture, Assiut University, Assiut, 71511 Egypt; 7 ERU Science & Innovation Center of Excellence, Egyptian Russian University, Badr City, Cairo 11829 Egypt

**Keywords:** Amylase, Cold-adapted, Peels, *Penicillium*, Potato, Solid-state, Waste

## Abstract

This study explores the utilization of potato peel waste as a substrate for producing cold-adapted amylase under solid-state fermentation (SSF) using *Penicillium goetzii* AUMC 498. Optimization through Box–Behnken design identified pH, incubation period, and beef extract concentration as key factors, achieving a maximum activity of 4.21 U/g dry substrate at pH 7 and 10 °C after 6 days. The enzyme was purified via two-column chromatography (Trilite MA12 and Sephacryl S 200), resulting in a 63.6-fold increase in purity and a molecular weight of 51.91 kDa confirmed by SDS-PAGE. The purified amylase exhibited optimal activity at pH 5 and 25 °C, with sorghum starch as the preferred substrate (594.68 U/mg). Activity was significantly enhanced by MnSO₄ and ZnSO₄, while NiCl₂, SDS, and EDTA inhibited it. Kinetic constants for pure amylase were determined for six types of starch: soluble starch, sorghum starch, oat starch, maize starch, wheat starch, and rice starch. The Km values were 575.75 mM, 59.1 mM, 149.27 mM, 195.9 mM, 81.44 mM, and 37.48 mM, respectively. These findings demonstrate the effective conversion of potato peel waste into a value-added cold-adapted biocatalyst with desirable catalytic and stability characteristics. The ability of the enzyme to function efficiently at low temperatures highlights its strong potential for use in energy-efficient industrial processes, particularly in the food, detergent, textile, and starch-processing industries. Moreover, the utilization of agro-industrial waste as a fermentation substrate provides an environmentally sustainable and economically attractive approach, reinforcing the relevance of this study to waste valorisation strategies and green biotechnological applications.

## Introduction

The starch polymer is biodegraded into glucose, maltose, maltotriose, and dextrin by amylases, particularly α-amylase (EC 3.2.1.1), β-amylase (EC 3.2.1.2), and γ-amylase (EC 3.2.1.3) [1, 2]. In 2018, the enzyme market was valued at around US$ 5.5 billion, and by 2025, it is projected to surpass US$ 7.0 billion [3]. One third to one half of the enzyme market worldwide is devoted to amylases, placing them among the most important enzymes [2]. Among the many fields that make heavy use of them are analytical chemistry, biotechnology, medicine, pharmaceuticals, and the food and beverage, textile, bioethanol, and paper industries [2, 4–6].

Cold-adapted enzymes can continue catalyzing even when temperatures drop to 0 °C [7]. Cold-adapted enzymes differ from mesophilic and thermophilic ones in a number of ways, including their low reaction energy (the ideal catalytic temperature is usually between 20 and 45 °C), stronger substrate affinity (which can lower the activation energy of enzymatic reactions), and poor thermal stability at high temperatures (they lose over half of their activity after 10 min at 50–60 °C or several hours at 37 °C [8–10]. Due to their abundant source, short production cycle, high yield, and facile separation, microbial cold-active enzymes have garnered considerable interest. These enzymes can also be easily controlled reaction conditions, and their purification and monitoring of manufacturing processes is simplified [11, 12]. These enzymes have been studied and utilized in numerous fields, including as food processing, detergent production, bioremediation, environmental preservation, straw resourcing, and fundamental molecular biology [13].

Cold-adapted enzymes are characterized by their ability to catalyze biochemical reactions efficiently at low temperatures, making them highly attractive for biotechnological and industrial applications that require mild processing conditions. Their effective catalytic activity at reduced temperatures enables significant energy savings, lowers operational costs, and minimizes thermal damage to heat-sensitive substrates, which is particularly advantageous in large-scale industrial processes [7, 14]. As a result, cold-active enzymes have gained increasing interest in sectors such as food processing, detergents, textiles, starch conversion, and environmental biotechnology, where low-temperature operation is desirable or essential. These properties position cold-adapted amylases as valuable biocatalysts for the development of energy-efficient and sustainable industrial processes [7, 14–16].

Despite the well-recognized industrial advantages of cold-adapted enzymes, their large-scale biotechnological application is highly dependent on the availability of low-cost, renewable substrates that can support economically viable enzyme production processes. In this context, potato peels constitute one of the most abundant (70,000 and 140,000 tons annually) agro-industrial waste by-products generated by the potato-processing industry. Large quantities of potato peel waste are produced annually, creating environmental and disposal challenges [17, 18]. Owing to their rich content of starch, non-starch carbohydrates, proteins, and other nutrients, potato peels represent an inexpensive and renewable substrate that can effectively support microbial growth and enzyme production [19]. The valorization of this waste material as a fermentation substrate therefore offers a sustainable and economically attractive strategy for producing value-added biotechnological products such as cold-adapted amylases [20–22].

Although cold-adapted enzymes have attracted growing industrial interest for their suitability in energy-efficient, low-temperature bioprocesses, studies on cold-adapted amylases remain relatively scarce in comparison with mesophilic and thermophilic enzymes [23]. Moreover, most existing studies focus on bacterial sources, while fungal cold-adapted amylases are comparatively underexplored, despite their known advantages in secretion capacity and industrial applicability. In addition, there is a clear lack of studies integrating the production of cold-adapted amylases with low-cost, renewable agro-industrial wastes under solid-state fermentation conditions, which are critical for improving economic feasibility and sustainability [16, 24]. This gap highlights the need to identify new fungal producers capable of generating cold-adapted amylases using inexpensive waste substrates, and to comprehensively characterize their biochemical properties for potential industrial applications. Despite the recognized potential of cold-adapted amylases for energy-efficient industrial processes, studies focusing on their production by *Penicillium* species remain particularly limited. Most reports on fungal cold-active amylases have centered on a narrow range of psychrotolerant genera, with *Penicillium* being comparatively underexplored despite its well-known metabolic versatility and secretion capacity. Furthermore, most existing studies have employed soluble or commercial starches as substrates under submerged fermentation conditions, leaving a clear gap in knowledge regarding the use of low-cost, renewable lignocellulosic or agro-industrial residues—such as potato peels—for cold-adapted amylase production by *Penicillium* strains under solid-state fermentation (SSF). SSF offers distinct advantages for waste valorization, including lower water consumption, higher volumetric productivity, and closer mimicry of the natural fungal habitat [25], yet its application for cold-adapted amylase production from *Penicillium* species using real agro-industrial waste has seldom been investigated. This gap is particularly notable given that *Penicillium* species are known to thrive on complex plant-based materials and are efficient secretors of hydrolytic enzymes.

This study aimed to explore a novel and integrated approach based on three central pillars of biotechnological innovation: (i) the production of a cold-adapted amylase capable of efficient catalysis at low temperatures, which offers significant energy savings for industrial processes; (ii) the use of *P. goetzii* AUMC 498 as a fungal producer, a species rarely investigated for cold-active enzyme production despite its metabolic versatility; and (iii) the valorization of potato peel waste as a low-cost, renewable substrate under solid-state fermentation (SSF) conditions, an environmentally sustainable strategy that simultaneously addresses agro-industrial waste management. Specifically, this work aimed to optimize the production parameters using response surface methodology, utilize potato peels as the sole substrate for amylase production under SSF, and subsequently purify and characterize the resulting cold-adapted amylase for its biochemical properties and industrial potential.

## Materials and methods

### Potato peel material

Sample of potato peels (5 kg) was collected as waste from the peeling machine unit at a potato chip manufacturing facility in Assiut Governorate, Egypt. The collected peels were manually cleaned to remove unwanted debris, filtered to eliminate excess water, and thereafter stored in polyethylene bags in the freezer at − 25 °C until needed.

### Fungal strain

*Penicillium goetzii* AUMC 498 was shown to be quite effective in previous study that screened for cold-active amylase [26]. After 7 days at pH 7.0 and 10 °C, with beef extract as the nitrogen supplement, the strain showed its peak cold-active amylase activity. To maximize the cold-active amylase yield, this study was directed to enhance the production of the cold-active amylase under solid state fermentation conditions utilizing response surface methodology (RSM).

### Morphological identification of *Penicillium goetzii* AUMC 498

*Penicillium goetzii* AUMC 498 used in this study was morphologically identified using Czapek’s agar (CZ; [27], malt extract agar (MEA; [28], and Czapek’s yeast autolysate agar (CYA; [29]. Inoculations were made with spore suspensions suspended in a 0.2% agar and 0.05% Tween 80 solution [30]. In a three-point design, plates were infected with an inoculum size of 1.0 µL/spot using a micropipette. After that, the plates were incubated in the dark at 25 °C for 7 days. The CYA culture’s microscopic characteristics were examined.

### Molecular confirmation of the potent strain’ identification

Following the procedure outlined by Moubasher et al. [31], DNA isolation of the *Penicillium goetzii* AUMC 498 was carried out. The internal transcribed spacer (ITS) region was amplified using the universal primers ITS1 and ITS4 [32]. PCR procedures were carried out at SolGent Company (Yuseong-Gu, Daejeon, South Korea) according to [33]. ITS sequence of the *Penicillium* sp. AUMC 498 in this study along with the most similar sequences in GenBank were aligned by MAFFT (version 6.861b) [34]. *Penicillium glabrum* CBS 125543 served as outgroup. BMGE [35] optimized alignment gaps and parsimony uninformative characters. MEGA X (version 10.2.6) [36] conducted the Maximum-likelihood (ML) and Maximum parsimony (MP) analyses, using a heuristic search of 1000 replications [37]. Akaike information criterion (AIC) as implemented in Modeltest 3.7 selected the best model of nucleotide substitution [38].

### Improvement of amylase production using response surface methodology (RSM)

The experiment was performed in 500 mL Erlenmeyer flasks, each containing 10 g of fresh potato peels. The substrate was individually hydrated with 10 mL of sucrose-free Cz broth supplemented with 0.1% soluble starch solution. Box–Behnken (BBD) of response surface methodology (RSM) was used to optimize three independent variables in this experiment: incubation period, pH, and beef extract. These three factors were selected based on the results of our preliminary one-factor-at-a-time experiments (data not shown), which indicated that pH, incubation period, and nitrogen source (beef extract) concentration exerted the most significant individual effects on cold-active amylase production by *P. goetzii* AUMC 498. Temperature was maintained constant at 10 °C throughout the optimization to specifically target cold-adapted enzyme production, while other medium components were kept at fixed concentrations based on preliminary screening that showed minimal influence on enzyme yield. This experimental logic prioritizes factors with the highest potential for interactive effects, making them suitable for response surface methodology. All experimental flasks were incubated at 10 °C under solid-state fermentation conditions during the optimization process. Variables were evaluated at three code levels (− 1, 0, + 1) to study their effect on amylase yield. The experimental plans are detailed in Table 1. The design expert program was used to build fifteen separate experiments, each with a single central point. For each component to achieve its full potential in terms of amylase activity, point prediction methods were used. Iterative techniques, system identification, and parameter estimation were applied to the model to produce better accuracy and reliable simulation outcomes. A polynomial equation of second order that has been generated is presented in Eq. (1):

1 $${\mathrm{Y}}={\upbeta _{\mathrm{o}}}+\sum {{\upbeta _{\mathrm{i}}}{{\mathrm{X}}_{\mathrm{i}}}} +\sum {{\upbeta _{{\mathrm{ij}}}}{{\mathrm{X}}_{\mathrm{i}}}{{\mathrm{X}}_{\mathrm{j}}}} +\sum {{\upbeta _{{\mathrm{ii}}}}{{\mathrm{X}}_{\mathrm{i}}}^{{\mathrm{2}}}} $$

where Y is the predicted response, β_0_ is the model constant; X_i_ are independent factors (incubation period, pH, and beef extract); β_i_ and β_ii_ are coefficients. To determine the significance of the model terms, the data obtained from the BBD were subjected to ANOVA analysis.

**Table 1:** The influencing factors and their levels in Box–Behnken design BBD

Factor	Symbol	Code level		
		− 1	0	+ 1
pH	A	6.5	7	7.5
Incubation period (day)	B	6	7	8
Beef extract (g/L)	C	1.5	2	2.5

### Production of amylase under SSF

Using solid-state fermentation conditions and optimal fermentation parameters, amylase production was conducted at 10 °C in 1000 mL Erlenmeyer flasks, each containing 50 g of fresh potato peels. The initial moisture content of the substrate was adjusted to approximately 70% (w/w) by hydrating the peels with 50 mL of sucrose-free Cz broth supplemented with 0.1% soluble starch solution at the optimal pH level. The flasks were inoculated with 1 × 10⁶ spores per gram of dry substrate (spores/gds) using a spore suspension prepared from 7-day-old cultures of *P. goetzii* AUMC 498 grown on Czapek agar. The inoculum density was determined using a hemocytometer. After inoculation, the flasks were loosely plugged with cotton wool to allow passive aeration and incubated under static conditions without forced aeration. Following incubation under optimal fermentation conditions, the cell-free supernatant was collected using centrifugation for 10 min at 10,000 rpm and 4 °C.

### Amylase assay and protein estimation

Following the incubation period, the cell-free supernatant was acquired using centrifugation (10,000 rpm at 4 °C for 10 min) and utilized as the amylase source. Amylase activity was determined by combining 0.5 mL of the supernatant with 0.5 mL of 1.0% starch (produced in 50 mM phosphate buffer at pH 7.0). The reaction mixture was incubated for 15 min at 10 °C (Bailey et al. 1992). The reaction was subsequently terminated by adding 2 mL of 3, 5-dinitrosalicylic acid (DNS) and boiling the reaction mixture in a water bath for 10 min (Miller 1959). Following cooling, the color absorbance was measured at 540 nm utilizing a UV-Visible spectrophotometer (Pg Instrument; Model: T80+; UK). One unit of amylase activity is defined as the quantity of enzyme necessary to liberate one µmol of glucose per mL per minute under standard test conditions. Total protein was quantified using Lowry’s method [39], with bovine serum albumin (BSA) serving as the standard.

### Precipitation and dialysis of amylase

Three volumes of cold absolute ethyl alcohol (− 25 °C) were employed to isolate the enzyme at 4 °C. The extracted protein was solubilized in citrate buffer (pH 5), subjected to dialysis twice for 2 h at ambient temperature (cutoffs: 12–14 kD), and subsequently refrigerated overnight at 4 °C to eliminate salts and other small molecules [9]. The dialyzed protein was further concentrated using a Lyophilizer (VirTis, model #6KBTES-55, NY, USA) and employed in the purification procedures.

### Purification of amylase

#### Trilite MA 12 column

Trilite MA 12 anion exchange resin, pre-activated with 0.5 M NaOH for 60 min, was packed into a glass column measuring 60 cm × 2.4 cm, with a bed volume of 200 cm³. A 10.0 mL sample was loaded into the gel after the equilibration with citrate buffer (pH 5). The enzyme was eluted using 100 mM citrate buffer (pH 5) at NaCl concentrations of 0, 0.1, 0.25, 0.5, 1.0, and 1.5 M. The column flow rate was modified to 0.25 mL/min, and the fractions were 5.0 mL in volume. The amylase activity and protein content were determined, and the most active fractions were pooled, concentrated, and subsequently employed in further purification.

#### Sephacryl S 200 column

A glass column (40 cm × 2.4 cm; bed volume 100 cm³) was packed with Sephacryl S 200 size exclusion gel. The enzyme was eluted with citrate buffer (pH 5) following the loading of the enzyme sample (5.0 mL) onto the column. Amylase activity and protein content were assessed in 5.0 mL fractions. The most active portions were collected and lyophilized.

### SDS-PAGE

A RIPA lysis solution comprising 1 µg/mL of leupeptin and aprotinin, 0.5 mM of phenylmethylsulphonyl fluoride (PMSF), 1 mM of sodium hydroxide, and 5 mM of sodium fluoride was utilized to solubilize protein samples, including both crude extracts and purified forms. The buffer comprised 50 mM Tris-HCl (pH 7.6), 5 mM EDTA, 150 mM sodium chloride, 0.5% NP-40, and 0.5% Triton-X-100. Following the estimation of protein quantities via the Lowry technique [39], sample buffer was added to portions of protein samples and boiled for 5 min. In the wells, 5 µL of pre-stained protein marker and 30 µL of sample, approximately 30 µg/mL of protein, were added. The stacking gel underwent electrophoresis at 60 V, while the separating gel was subjected to 120 V, all during cooling of the gel. The gel was extracted post-electrophoresis and subsequently stained with Coomassie blue for 30 min. The staining was permitted to remain overnight.

### Impact of pH, temperature and addition of some chemicals on amylase activity

pH values from 3 to 10 and temperatures from 10 to 50 °C were used to study the activity of pure amylase. A 100 µL of the pure amylase and 900 µL of soluble starch, both mixed in a 50 mM buffer solution, made up the reaction mixture. The amylase activity was then determined after adding 2.0 mL of 3,5-dinitrosalicylic acid (DNS) to stop the reaction after 5 min. Addition of some chemicals such as NaCl, KCl, CaCl_2_, CoCl_2_, NiCl_2_, CuSO_4_, FeSO_4_, MgSO_4_, MnSO_4_, ZnSO_4_, EDTA, and SDS were tested in these solutions at 5 mM. Three separate runs of each experiment were conducted.

### Determination of the kinetic constants (Km and Vmax)

The enzyme activity of the pure cold-active amylase was measured at various concentrations of starch (1–10 mg/mL) using a Lineweaver-Burk plot to estimate the values of Michaelis-Menten constant (Km) and maximum reaction velocity (Vmax) [40].

### Statistical analysis

After conducting the initial investigation three times, the mean and standard deviation (SD) were used to express all data. The statistical significance analysis was conducted [41], and the significance level was set at *p* ≤ 0.05.

## Results

### Morphological identification of *Penicillium goetzii* AUMC 498

**Fig. 1:**
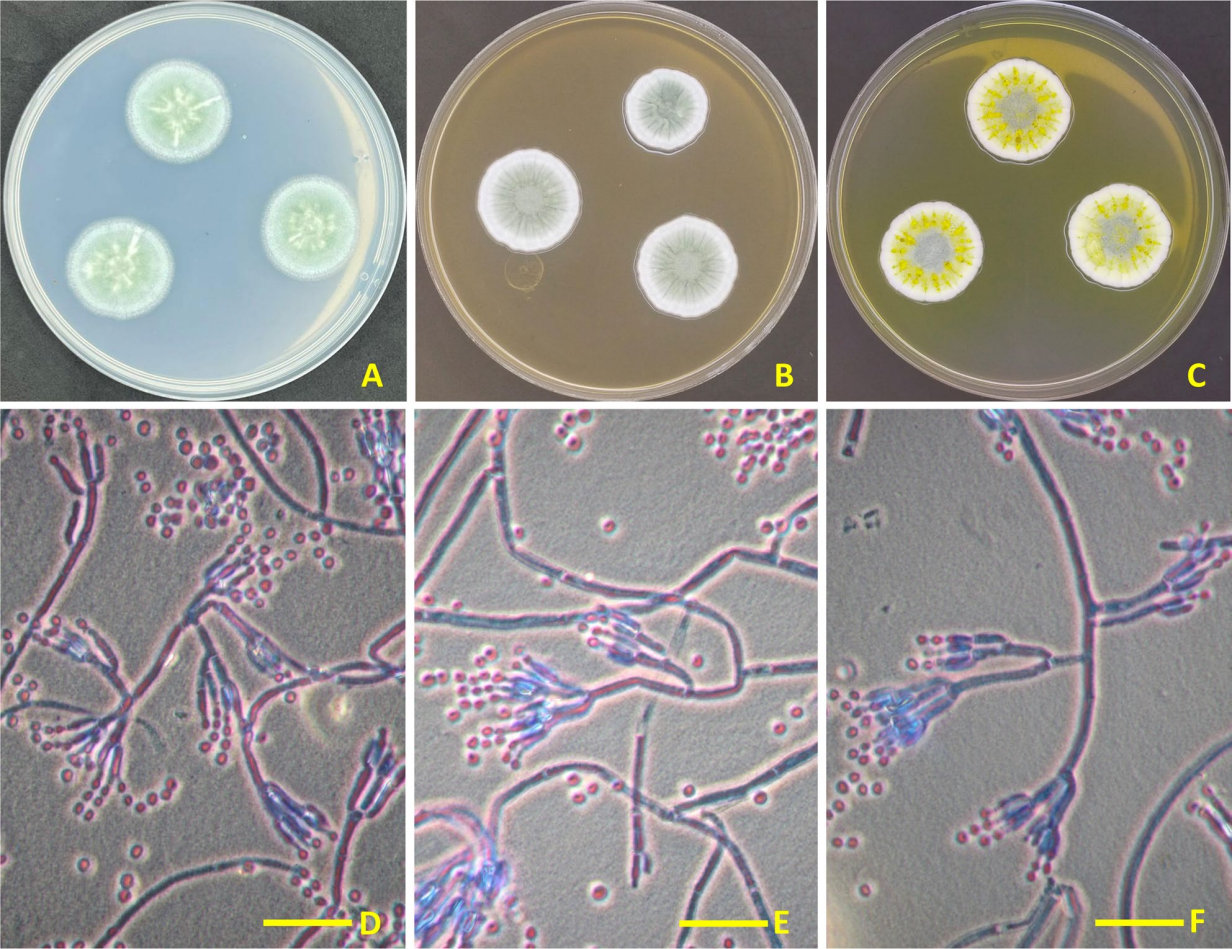
Morphological identification of *P. goetzii* AUMC 498. **A**–**C** Seven-day-old colonies at 25 °C on **A** Czapek agar (CZ), **B** malt extract agar (MEA), and **C** Czapek yeast autolysate agar (CYA). **D**–**F** Microscopic features showing smooth-walled conidiophore bearing metulae; Ampulliform phialides with developing conidial chains; Globose to subglobose, smooth conidia. All microscopic images captured at 1000× magnification with cotton blue in lactic acid (scale bar = 20 μm applies to **D**–**F** [42]

Colonies on MEA after 7 days at 25 °C floccose; sporulation variable, conidia grey green; mycelium white, conidia grey green. Conidiophores terverticillate to quarterverticillate, 200–400 × 2.5–3.5 μm, smooth walled. Metulae, 8–12(− 15) × 2.5–3.5 μm. Phialides ampulliform, 7–9(− 10) × 2–3 μm. Conidia broadly ellipsoidal, smooth, 2–2.5 × 2–3 μm (Fig. 1).

### Molecular identification of *Penicillium goetzii* AUMC 498

**Fig. 2:**
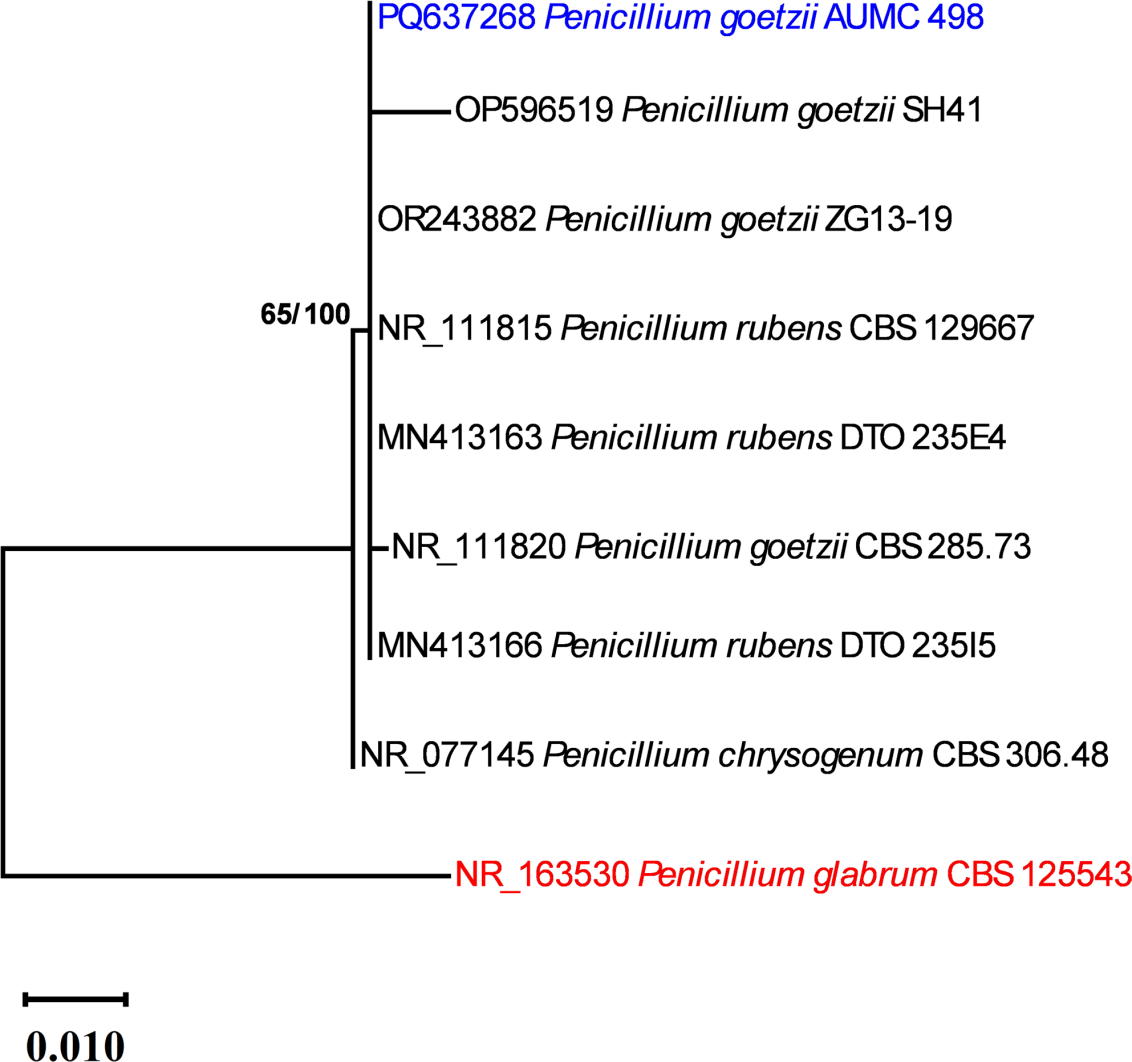
Maximum likelihood phylogenetic tree derived from a heuristic search (1000 replications) utilizing ML/MP analysis of ITS sequence of *P. goetzii* AUMC 498 (in blue) in relation to the most comparable species related to *Penicillium* in GenBank. Bootstraps indicating support values for ML/MP ≥ 50% are displayed adjacent to the respective nodes. The tree is rooted to *Penicillium glabrum* CBS 125,543 (in red)

The conclusive ITS dataset for this phylogenetic analysis comprised 585 characters obtained from the sequences of 9 species; 493 were accurately aligned, 44 were identified as variable, and one was classified as informative. The evolutionary relationship among taxa was effectively illustrated using Tamura’s 3-parameter model (T92). The Maximum Parsimony analysis produced ten trees. The maximum likelihood tree, illustrated in Fig. 2, is characterized by 47 steps and a maximum log likelihood of − 1037.14. The strain examined in this study was grouped with *P. goetzii* strains SH 41, ZG 13–19, and CBS 285.73 (type strain) on the same branch of the phylogenetic tree. Therefore, it was identified as *P. goetzii*. ITS sequence of *P. goetzii* AUMC 498 was deposited to GenBank under the accession number PQ637268 (Fig. 2).

### Optimization of the cold-active amylase using BBD

The previous experiment revealed that amylase activity was significantly influenced by three factors: pH, concentration of the beef extract, and incubation period. It’s ideal concentration and interaction were ascertained utilizing the BBD. A total of fifteen trials were conducted. Amylase activity reached a maximum of 4.21 U/gds after 6 days of incubation at pH 7 using 1.5 g/L beef extract as nitrogen source (run 9) and a minimum of 3.15 U/gds during run 13 (Table 2).

**Table 2:** Box–Behnken design (BBD) matrix with experimental factors and amylase activity under SSF by *P. goetzii* AUMC 498 at 10 °C

Run	pH	Incubation period (day)	Beef extract (g/L)	Amylase activity (U/gds)^*^
1	7.0	7.0	2.0	3.515
2	7.0	8.0	1.5	3.695
3	7.0	7.0	2.0	3.385
4	6.5	7.0	1.5	3.395
5	6.5	8.0	2.0	3.77
6	6.5	7.0	2.5	3.215
7	7.0	6.0	2.5	3.695
8	6.5	6.0	2.0	3.885
**9**	**7.0**	**6.0**	**1.5**	**4.21**
10	7.5	8.0	2.0	3.19
11	7.0	8.0	2.5	3.54
12	7.5	6.0	2.0	3.635
13	7.5	7.0	1.5	3.15
14	7.0	7.0	2.0	3.65
15	7.5	7.0	2.5	3.225

*U/gds = Units per gram of dry substrate. The maximum amylase activity (run 9) is shown in bold

The regression Equation of the BBD design for amylase activity (Eq. 2) coded units generated using the design expert software is presented as follows:

2 $$\begin{aligned} {\mathbf{Y1}} & ={\mathbf{0}}.{\mathbf{705}} - {\mathbf{0}}.{\mathbf{0266}}*{\mathbf{A}} - {\mathbf{0}}.{\mathbf{0307}}*{\mathbf{B}} - {\mathbf{0}}.{\mathbf{01956}}*{\mathbf{C}} - {\mathbf{0}}.{\mathbf{016632}}*{\mathbf{A}}*{\mathbf{B}} \\ & \quad +{\mathbf{0}}.{\mathbf{01796}}*{\mathbf{B}}*{\mathbf{C}} - {\mathbf{0}}.{\mathbf{04823479}}*{{\mathbf{A}}^{\mathbf{2}}}+{\mathbf{0}}.{\mathbf{0596842}}*{{\mathbf{B}}^2} \\ \end{aligned} $$

where Y1 is the amylase activity; A is the pH value; B is the incubation period (day); and C is the concentration of the beef extract.

The Model F-value of 7.05 indicated that the model was significant. The probability of obtaining an F-value of this magnitude due to random noise was about 0.87%. P-values below 0.0500 signify that the model terms are statistically significant. A, B, A², and B² were pertinent terms in the model. Values beyond 0.1000 signify that the model terms lack significance. The Lack of Fit F-value of 3.25 indicated that the Lack of Fit is not significant in comparison to the pure error. The probability of a Lack of Fit F-value of this magnitude arising from random variation was 14.22% (Table 3).

**Table 3:** ANOVA test results for amylase activity under SSF by *P. goetzii* AUMC 498

Source	Sum of squares	Df	Mean square	F-value	*P*-value	
Model	1.10	9	0.1217	7.05	0.0087	Significant
A-pH	0.1418	1	0.1418	8.22	0.0241	
B- Incubation period	0.1891	1	0.1891	10.96	0.0129	
C-Beef extract	0.0751	1	0.0751	4.35	0.0754	
AB	0.0272	1	0.0272	1.58	0.2494	
AC	0.0163	1	0.0163	0.9421	0.3641	
BC	0.0324	1	0.0324	1.88	0.2129	
A²	0.1990	1	0.1990	11.53	0.0115	
B²	0.4349	1	0.4349	25.20	0.0015	
C²	0.0116	1	0.0116	0.6694	0.4402	
Residual	0.1208	7	0.0173			
Lack of fit	0.0857	3	0.0286	3.25	0.1422	Not significant
Pure error	0.0351	4	0.0088			
Cor total	1.22	16				

**Fig. 3:**
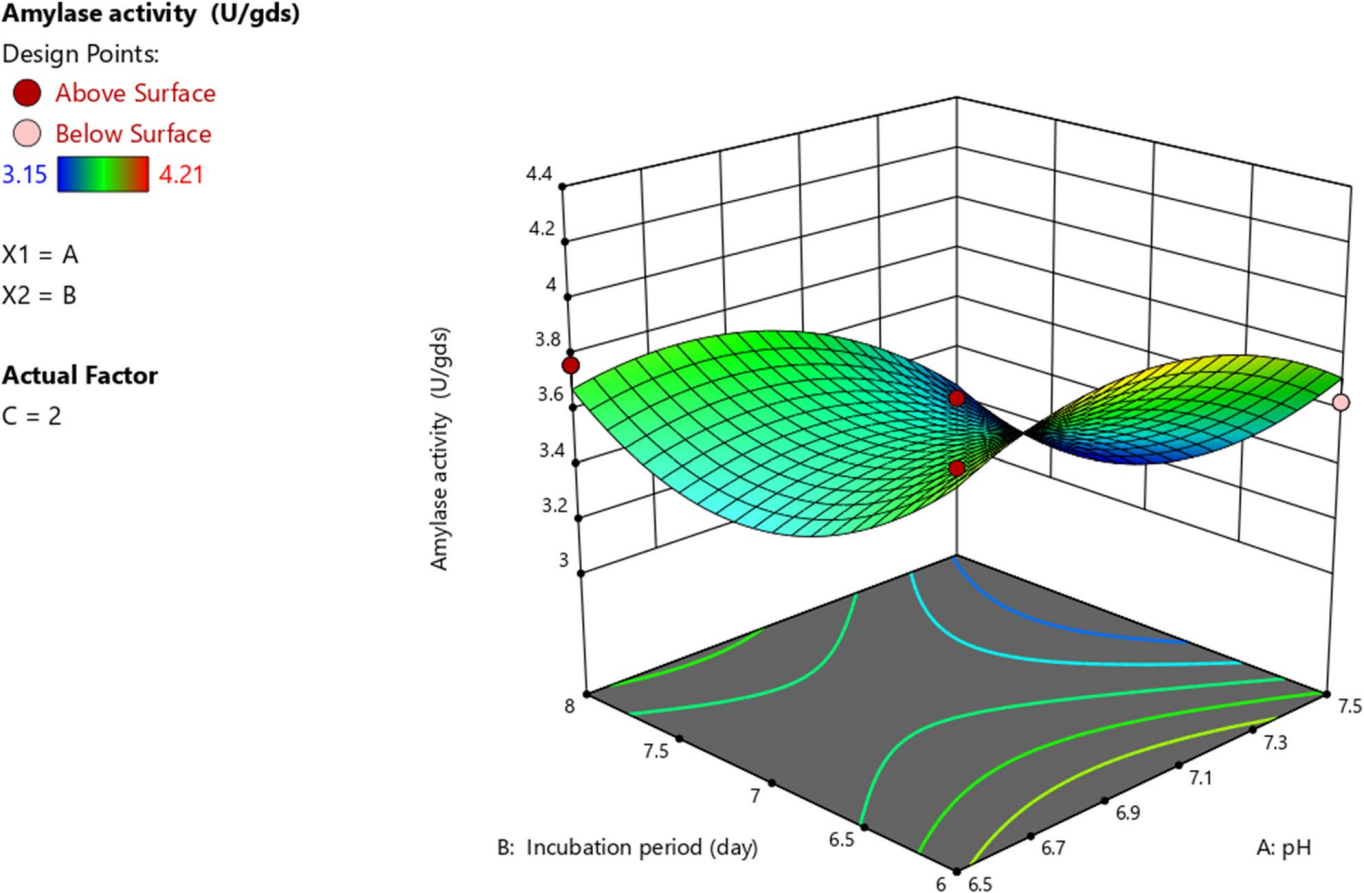
Response surface plot (3D) showing the interactive effect of pH and incubation period (days) on cold-adapted amylase activity (U/gds) produced by *P. goetzii* AUMC 498 under SSF. Beef extract concentration was held constant at 2.0 g/L. Amylase activity was assayed at 10 °C

**Fig. 4:**
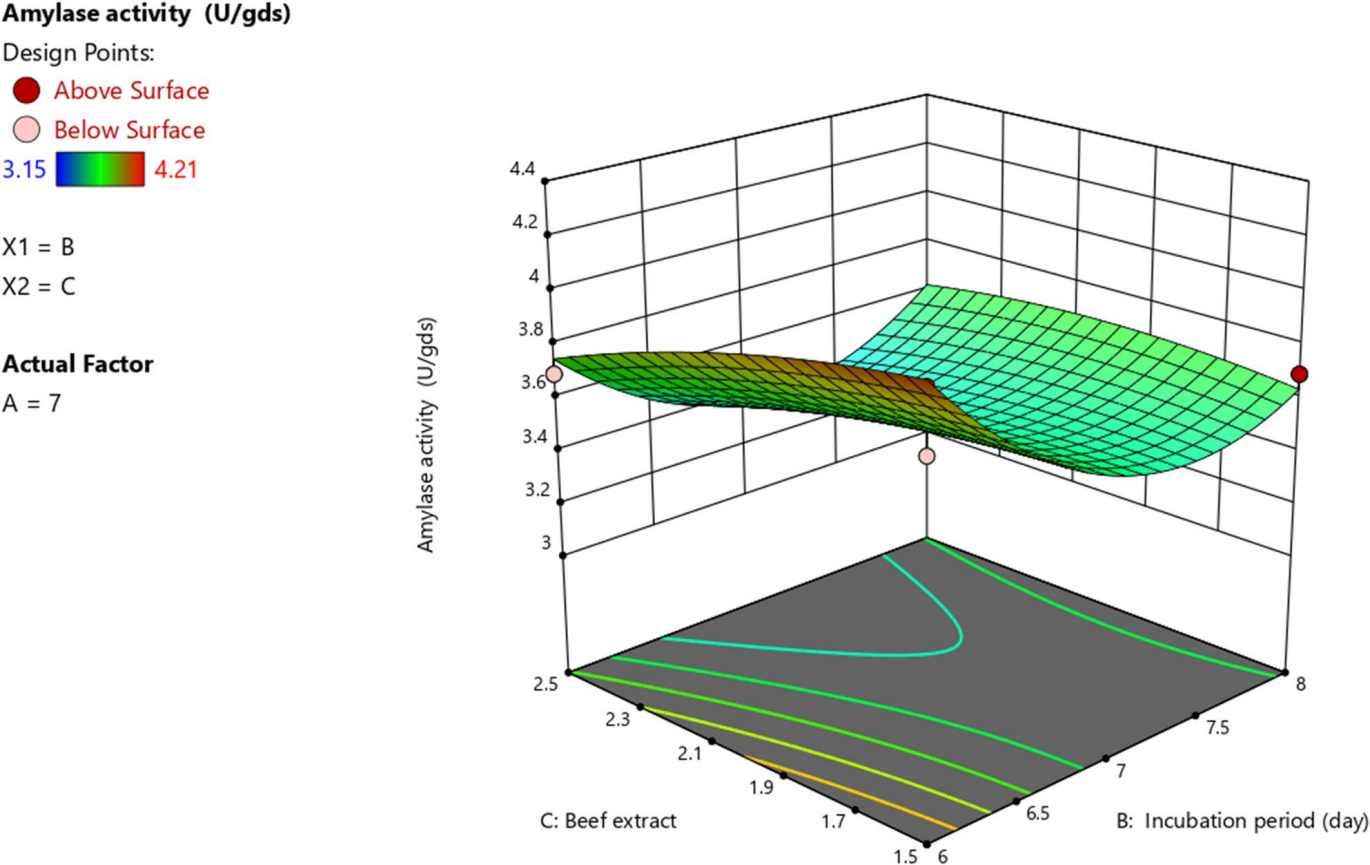
Response surface plot (3D) showing the interactive effect of beef extract concentration (g/L) and incubation period (days) on cold-adapted amylase activity (U/gds) produced by *P. goetzii* AUMC 498 under SSF. pH was held constant at 7.0. Amylase activity was assayed at 10 °C

Regression analysis was used to generate 3D surface plots to investigate the interaction between variables and identify the optimal value of each variable for achieving the highest amylase activity. In these plots, two parameters were varied while the remaining variables were held at their midpoint values. The results revealed that both pH and incubation period (Fig. 3), as well as beef extract and incubation period (Fig. 4) demonstrated a significant impact on amylase activity.

### Validation

To validate the regression model used in this study, an experiment using the parameters suggested by the RSM optimizer was conducted. The measured amylase activity (4.435 U/gds) was very close to the predicted value (4.1154 U/gds), respectively proving the accuracy of the selected model. With these results, the selected model was validated.

### Amylase purification

**Fig. 5:**
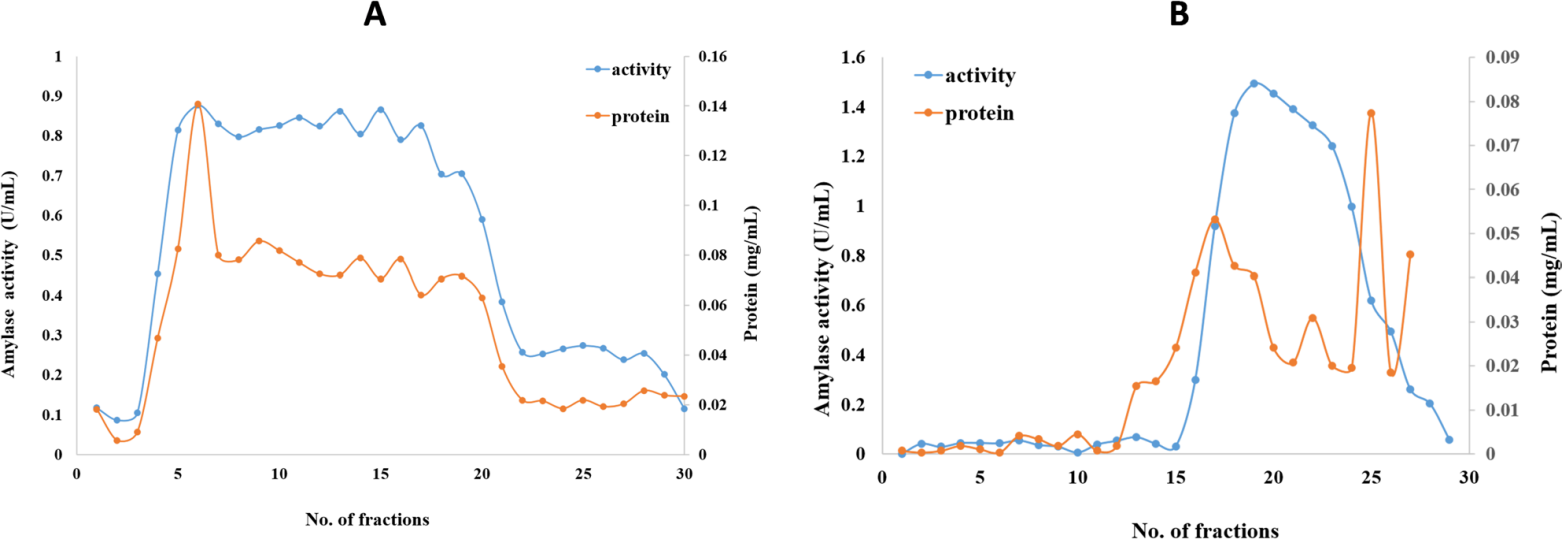
Elution diagram of cold adapted amylase produced by *P. goetzii* AUMC 498 using **A** Trilite MA 12 column as anion exchange chromatography and **B** Sephacryl S 200 size exclusion column

*Penicillium goetzii* AUMC 498 produced cold-adapted amylase under optimal fermentation conditions of 6 days of incubation at 10 °C and pH 7, using beef extract as the nitrogen source. Following the incubation period, the collected cell-free supernatant (2000 mL) underwent filtering, and the total protein was extracted using cold 100% ethyl alcohol. The partially purified amylase, following precipitation, dialysis, and lyophilization, was subsequently purified using anion exchange chromatography with a Trilite MA 12 column. The anion-exchange chromatography produced 18 pooled fractions (Fractions no. 4–21; Fig. 5A) with the greatest peaks of amylase and protein. After collecting and concentrating the most active fraction, the specific activity attained 14.3 U/mg. The most active fractions from the Trilite MA 12 column were collected and further purified utilizing the Sephacryl S 200 column. Gel filtration on Sephacryl S 200 produced pronounced peaks of amylase activity and protein concentration (Fractions no. 17–25; Fig. 5B). After two-column purification process resulted in a 63.6-fold increase in amylase purity and a yield of 5.9%, with a specific activity of 166 U/mg (Table 4).

**Table 4:** Purification profile of cold-adapted amylase produced by *P. goetzii* AUMC 498 under SSF utilizing potato peels after 6 days of incubation at pH 7.0 and 10 °C using beef extract as nitrogen source

Purification step	Volume (mL)	Activity (U/mL)	Protein (mg/mL)	Total activity (U)	Total protein (mg)	Specific activity (U/mg)	Yield (%)	Fold
Fermentation medium	2000	0.842	0.322	1684	644	2.61	100	1
Absolute ethanol	90	5.64	0.865	507.6	77.85	6.52	30.14	2.5
Trilite MA12	35	4.1	0.29	143.5	10.15	14.13	8.52	5.41
Sephacryl S 200	20	4.98	0.03	99.6	0.6	166	5.9	63.6

Amylase activity was assayed at 10 °C

### SDS-PAGE

**Fig. 6:**
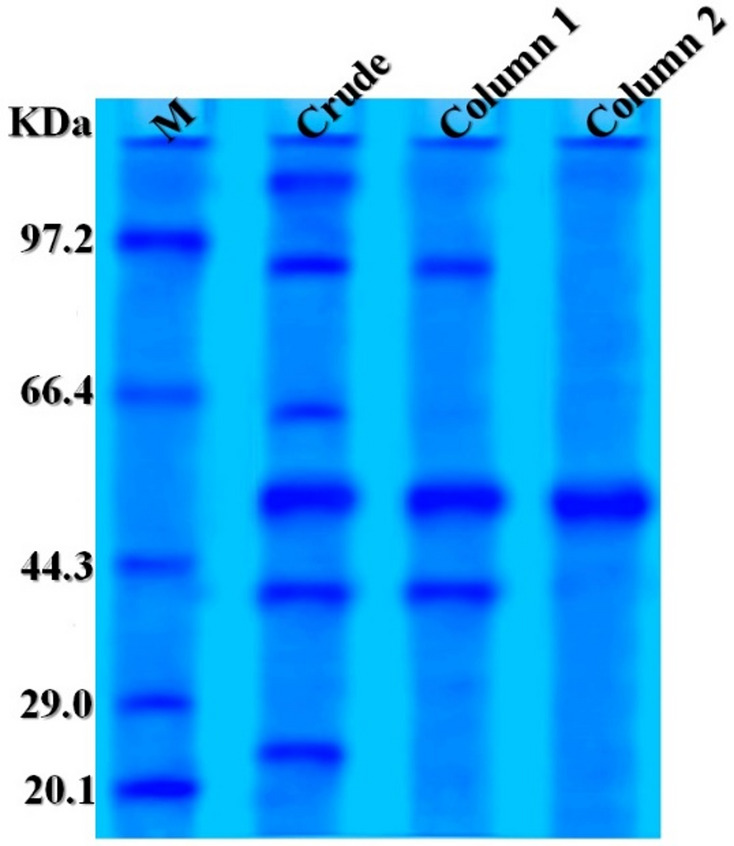
SDS-PAGE of amylase produced by *P. goetzii* AUMC 498. M: pre-stained marker. Lane 1: crude enzyme. Lane 2: Trilite MA 12 anion exchange column. Lane 3: pure amylase from Sephacryl S 200 column

The SDS-PAGE results indicated that the amylase produced by *P. goetzii* AUMC 498 was homogenous and completely pure. The molecular weight was ascertained to be 51.91 kDa (Fig. 6).

### Determination of optimal pH and temperature for pure amylase

**Fig. 7:**
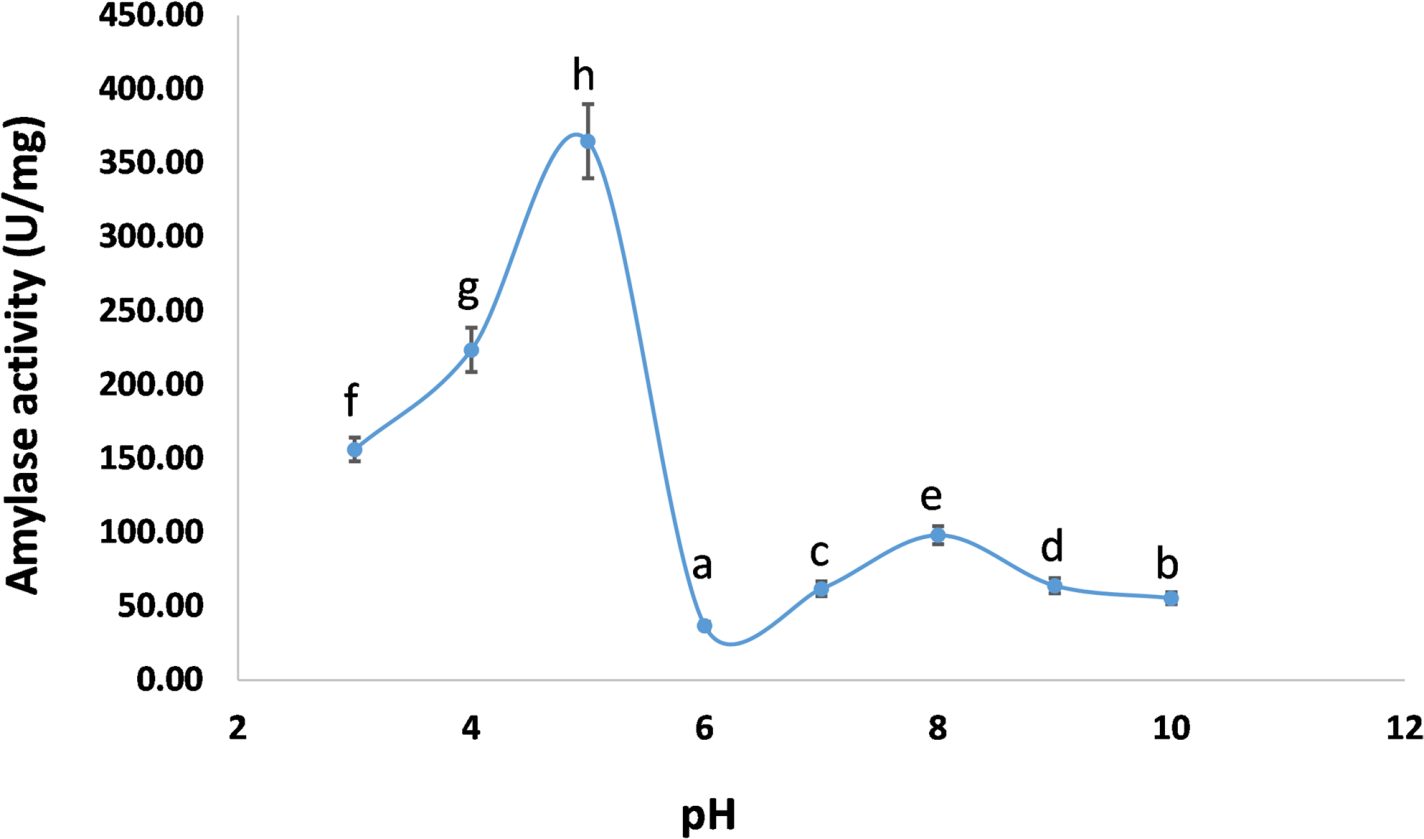
Effect of pH on the activity of the purified cold-adapted amylase from *P. goetzii* AUMC 498. Amylase activity was assayed at 20 °C using soluble starch (1% w/v) as substrate in 50 mM buffer systems: citrate buffer (pH 3–6), phosphate buffer (pH 7–8), and glycine-NaOH buffer (pH 9–10). The reaction mixture (0.5 mL enzyme + 0.5 mL 1% starch) was incubated for 15 min. Activity is expressed as U/mg protein. Results are means of three replicates ± SD. Different superscript letters (a–e) above the bars indicate statistically significant differences at *p* ≤ 0.05 (one-way ANOVA, Tukey’s post-hoc test). The optimal pH was 5.0, with an activity of 364.82 ± 25 U/mg

**Fig. 8:**
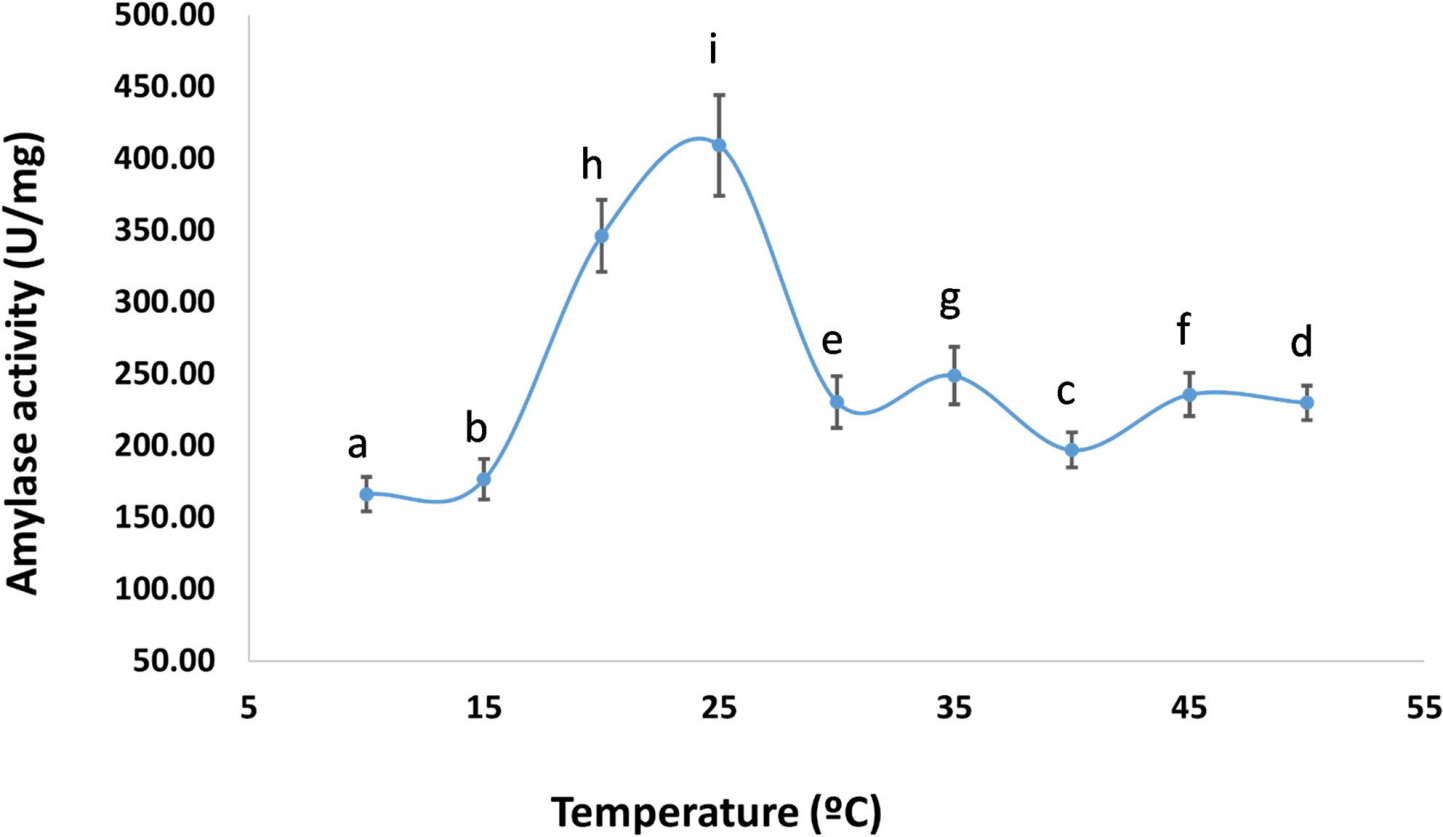
Effect of temperature on the activity of the purified cold-adapted amylase from *P. goetzii* AUMC 498. Amylase activity was assayed at the optimal pH of 5.0 using soluble starch (1% w/v) as substrate in 50 mM citrate buffer. The reaction mixture (0.5 mL enzyme + 0.5 mL 1% starch) was incubated for 15 min at temperatures ranging from 10 °C to 50 °C. Activity is expressed as U/mg protein. Results are means of three replicates ± SD. Different superscript letters (a–f) above the bars indicate statistically significant differences at *p* ≤ 0.05 (one-way ANOVA, Tukey’s post-hoc test). The optimal temperature was 25 °C, with an activity of 409.11 ± 35 U/mg. Notably, the enzyme retained 89.2% activity at 20 °C and 68.5% activity at 10 °C, highlighting its cold-adapted nature

At 20 °C, variation of pH from 3 to 10 showed that the pure amylase reached its optimal amylase activity at pH 5, recording 364.82 ± 25 U/mg, after which the activity gradually decreased from pH 6 to 10 (Fig. 7). The amylase activity increased further with temperature and reached a maximum value of 409.11 U/mg at 25 °C (Fig. 8).

### Substrate specificity

**Fig. 9:**
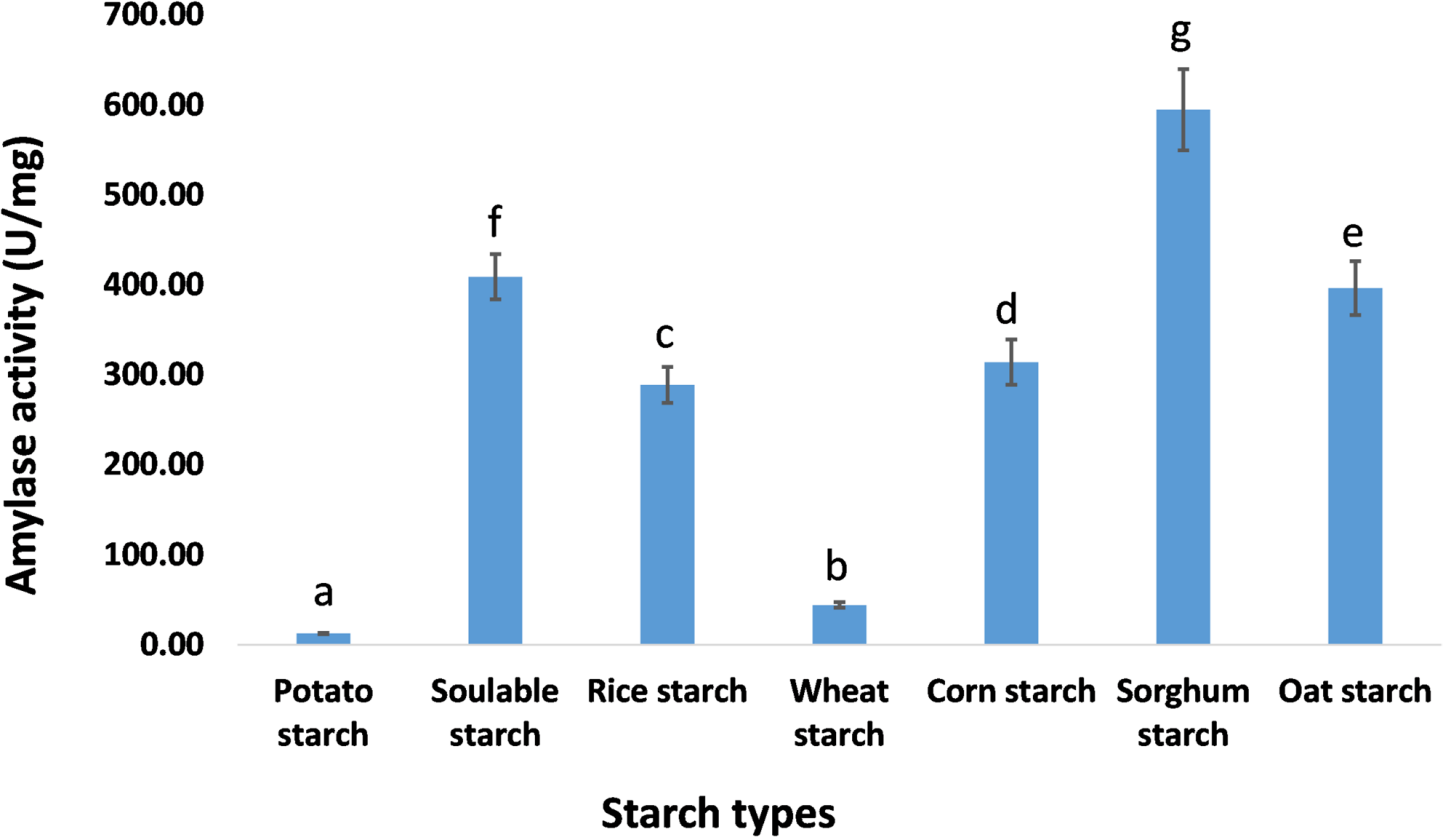
Substrate specificity of the purified cold-adapted amylase from *P. goetzii* AUMC 498. Amylase activity was assayed at the optimal pH of 5.0 (50 mM citrate buffer) and optimal temperature of 25 °C for 15 min. Seven different starch substrates were tested at a final concentration of 1% (w/v): soluble starch (positive control), sorghum starch, oat starch, maize starch, wheat starch, rice starch, and potato starch. Activity is expressed as U/mg protein. Results are means of three replicates ± SD. Different superscript letters (a–g) above the bars indicate statistically significant differences at *p* ≤ 0.05 (one-way ANOVA, Tukey’s post-hoc test). The enzyme showed the highest activity with sorghum starch (594.68 ± 45 U/mg), followed by soluble starch (409.11 ± 35 U/mg). The lowest activity was observed with potato starch (12.65 ± 0.85 U/mg), indicating strong substrate preference

At the optimum pH of 5 and temperature of 25 °C, the pure amylase exhibited varying degrees of amylase activity toward different starch substrates. The enzyme showed the highest amylase activity with sorghum starch (594.68 ± 45 U/mg), followed by soluble starch (409.11 U/mg), oat starch (396.45 ± 30 U/mg), maize starch (314.21 ± 25 U/mg), and rice starch (288.91 ± 20 U/mg). In contrast, the lowest amylase activity was observed with wheat starch (44.28 ± 3 U/mg) and potato starch (12.65 ± 0.85 U/mg) (Fig. 9).

### Effect of addition of some chemicals on the activity of pure amylase

The addition of various chemical compounds notably influenced the amylase activity of the purified enzyme. Among the tested additives, MnSO₄ and ZnSO₄ showed the strongest enhancement, increasing amylase activity to 635.22 ± 52 and 619.50 ± 50 U/mg, corresponding to 155.26% and 151.40% residual activity, respectively, compared with the control (409.11 ± 35 U/mg). Other compounds, including CoCl₂, NaCl, MgSO₄, FeSO₄, CaCl₂, KCl, and CuSO₄, also enhanced amylase activity to varying extents, whereas NiCl₂, SDS, and EDTA inhibited the enzyme activity (Table 5).

**Table 5:** Effect of various ions and inhibitors (at pH 5.0 and 25 °C) on the specific activity of the pure cold-active amylase produced by *P. goetzii* AUMC 498

Chemical compounds	Amylase activity (U/mg)	Residual activity (%)
Control	409.11 ± 35^d^	100 ± 8.56^d^
NaCl	532.99 ± 40^j^	130.28 ± 9.78^j^
KCl	494.18 ± 42^f^	120.8 ± 10.27^f^
CaCl_2_	507.12 ± 45^g^	123.95 ± 10.99^g^
MgSO_4_	528.44 ± 45^i^	129.16 ± 10.99^i^
MnSO_4_	635.22 ± 52^m^	155.26 ± 12.71^m^
ZnSO_4_	619.5 ± 50^l^	151.4 ± 12.22^l^
FeSO_4_	513.85 ± 45^h^	125.6 ± 10.99^h^
CuSO_4_	459.9 ± 35^e^	112.4 ± 8.56^e^
NiCl_2_	300.48 ± 22^b^	73.44 ± 5.38^b^
CoCl_2_	569.6 ± 45^k^	139.23 ± 10.99^k^
SDS	240.53 ± 18^a^	58.8 ± 4.39^a^
EDTA	316.87 ± 22^c^	77.45 ± 5.38^c^

Results are presented as mean ± SD of three replicates. Superscript letters (a–m) within the same column indicate statistically significant differences among treatments as determined by one-way ANOVA followed by Tukey’s post-hoc test (*p* ≤ 0.05). Treatments sharing the same superscript letter are not significantly different from each other; treatments with different superscript letters differ significantly

### Determination of Km and Vmax

Kinetic constants for pure amylase were ascertained for six varieties of starch: soluble starch, sorghum starch, oat starch, corn starch, wheat starch, and rice starch. The Km values were 575.75, 59.1, 149.27, 195.9, 81.44, and 37.48 mM, respectively. The Vmax values were 1250, 136.98, 108.7, 156.25, 5, and 46.5 µmol/min, respectively (Table 6).

**Table 6:** Kinetic parameters of substrate specificity of the pure amylase

Substrate	K_m_ (mM)	V_max_ (µmol/min)
Soluble starch	575.75	1250
Sorghum starch	59.1	136.98
Oat starch	149.27	108.7
Corn starch	195.9	156.25
Wheat starch	81.44	5.0
Rice starch	37.48	46.5

## Discussion

Amylases are essential enzymes that catalyze the hydrolysis of polysaccharides, including starch, into simple sugar constituents. These enzymes have demonstrated significant potential across diverse industrial applications, including dairy, baking, brewing, detergent, textile, and paper sectors, as well as starch saccharification processes. Contemporary technological advancements have further expanded the utility of amylases into clinical, pharmaceutical, and analytical chemistry domains [43]. Recent investigations into extracellular amylase production by various microorganisms have garnered considerable interest due to their potential as biotechnological sources of industrially relevant enzymes [42–46].

The present study represents the first demonstration of amylase production by *Penicillium goetzii* AUMC 498 utilizing potato peel waste as a substrate under solid-state fermentation (SSF). Potato (*Solanum tuberosum*) constitutes the fourth most cultivated crop globally following rice, wheat, and maize, representing an integral component of human dietary patterns worldwide. Although peeling is an essential preliminary step in potato processing for food applications, it inevitably generates substantial by-product streams. Industrial processing annually produces approximately 70,000–140,000 metric tons of potato peels [47]. The urgent necessity to reduce feedstock waste and alleviate environmental pressure associated with potato peel disposal has underscored the growing importance of complete utilization of this waste stream within potato processing industries [48]. Historically, this waste material has been converted into low-value products including animal feed, fertilizer, or biogas. However, recent investigations have revealed multiple valorization strategies for potato peels that enhance their economic value, particularly within food processing, phytopharmaceutical applications, and biosynthetic sectors encompassing lactic acid production, phenolic acid extraction, and ultrasonic-assisted recovery of steroidal alkaloids [48]. Despite its indigestible fibrous nature [49], the abundant starch content, non-starch polysaccharides, lignin, polyphenols, protein, and modest lipid content of potato peel waste render it a valuable low-cost by-product, serving as an economical and valuable base material for fermentation operations and extraction of valuable products including biopolymers, natural antioxidants, and dietary fiber [50–52].

The findings of this investigation demonstrated that amylase activity was significantly influenced by pH, beef extract concentration, and incubation period. The optimal concentrations and interactions among these variables were determined utilizing Box–Behnken design (BBD). Medium optimization is essential for advancing fermentation technology; nevertheless, research employing multi-factorial design for optimization of medium composition—particularly for fungal amylase production under SSF conditions—remains limited. The optimization of production conditions in this study revealed that *P. goetzii* AUMC 498 is a promising amylase producer, achieving maximum amylase activity of 4.21 U/gds after 6 days of incubation at pH 7.0 utilizing beef extract as nitrogen source. The majority of research on fungal α-amylase production has focused on mesophilic species with the objective of identifying superior fungal strains for industrial-scale production and establishing optimal growth conditions [1, 46, 53, 54]. *Aspergillus* and *Penicillium* represent the two predominant fungal genera for amylase synthesis [1, 6, 42, 46, 53–56].

The application of advanced chemometric tools for enzyme production optimization has recently been demonstrated by Araujo et al. [57], who used a constrained mixture design combined with a desirability function to simultaneously optimize the production of three cellulolytic enzymes (endoglucanase, exoglucanase, and β-glucosidase) by *Penicillium roqueforti* ATCC 10110 under SSF. Their desirability value of 0.84 indicated successful multi-response optimization, a strategy that could be adapted for the simultaneous production of multiple starch-hydrolyzing enzymes (e.g., α-amylase, glucoamylase, pullulanase) from *P. goetzii* AUMC 498 using potato peel waste.

The optimal conditions for amylase production vary considerably among fungal species, reflecting their diverse ecological niches and metabolic adaptations. For example, mesophilic *Penicillium* species typically exhibit peak amylase production at 25–35 °C under SSF, with yields ranging from 300 to 1012 U/gds depending on the substrate and nitrogen source [56, 58, 59]. In contrast, our study achieved maximum activity of 4.21 U/gds at 10 °C—a temperature at which most mesophilic fungi show negligible enzyme production. This substantially lower yield is expected, as cold-adapted enzyme production prioritizes catalytic efficiency at low temperatures over volumetric productivity, a trade-off that is economically justified by the significant energy savings in downstream applications [7]. Among the few studies reporting cold-active fungal amylases, *P. citrinum* AUMC 576 produced 9.92 U/mL at 25 °C under submerged fermentation, while our previous screening of *P. goetzii* AUMC 498 in submerged culture yielded 3.7 U/mL at 10 °C [42]. The present study therefore demonstrates, for the first time, the feasibility of producing a cold-adapted amylase from *P. goetzii* using potato peel waste under SSF at 10 °C. The novelty lies not in achieving the highest absolute yield, but in the successful integration of three underutilized pillars—a cold-adapted enzyme, a rarely studied *Penicillium* species, and an agro-industrial waste substrate under SSF conditions—offering a sustainable, energy-efficient alternative to conventional mesophilic production. The production of α-amylase under SSF using agro-industrial wastes has also been investigated by Pereira et al. [4], who used cassava residue as substrate for *Aspergillus niger* at 30 °C. While they achieved a shorter fermentation time (35 h) compared to our 6 days, their enzyme exhibited mesophilic characteristics with optimal activity at 50 °C, rendering it unsuitable for low-temperature applications. In contrast, our cold-adapted amylase from *P. goetzii* AUMC 498, produced at 10 °C, maintained high catalytic activity at 25 °C and retained 68.5% activity at 10 °C.

The present study achieved a 63.6-fold purification of the cold-adapted amylase through two-column chromatography—Trilite MA12 (anion exchange) followed by Sephacryl S 200 (size exclusion)—and SDS-PAGE analysis confirmed enzyme homogeneity and complete purity as a single band. The molecular weight was determined to be 51.91 kDa. Peak amylase activity was recorded at pH 5.0 and 25 °C, with a specific activity of 409.11 U/mg. α-Amylase typically exhibits optimal function at slightly acidic to alkaline pH levels, demonstrating stability within a pH range of 5.0–8.0. At acidic pH, the elevated concentration of hydrogen ions may obstruct enzyme active sites [60]. In this context, α-amylase from *P. camemberti* PL21 was produced using orange waste. Ammonium sulfate precipitation followed by column chromatography using Sephadex G-100 and DEAE-Sepharose CL-6B resulted in a 38.5-fold purification of α-amylase, with optimal pH and temperature established as pH 6.0 and 30 °C, respectively [61]. α-Amylase was produced using soybean meal in an SSF process by *Aspergillus oryzae* S2. Using acetone precipitation and size exclusion chromatography, the enzyme was isolated, exhibiting peak activity at pH 5.6 and 60 °C [62]. The partly purified amylase from *Bacillus subtilis* demonstrated its maximum activity of 13.14 U/mg at pH 7.1 and 40 °C [63]. Furthermore, Pereira et al. [4] employed aqueous two-phase systems (ATPS) for enzyme pre-concentration, achieving a selectivity of 4.974 with PEG 2000. While ATPS offers scalability and low cost, our two-column chromatography (Trilite MA12 and Sephacryl S200) achieved a 63.6-fold purification with a final specific activity of 166 U/mg, representing a higher purity suitable for detailed biochemical characterization. An integrated approach combining ATPS for initial capture followed by size exclusion chromatography could potentially improve our current yield of 5.9% while maintaining product purity.

At an optimal pH of 5 and a temperature of 25 °C, the pure amylase in this investigation hydrolyzed various types of starch, though to differing degrees. It exhibited maximum activity using sorghum starch as the substrate, with a specific activity of 594.68 ± 45 U/mg. Soluble starch yielded 409.11 U/mg, followed by oat starch at 396.45 ± 30 U/mg, maize starch at 314.21 ± 25 U/mg, and rice starch at 288.91 ± 20 U/mg. The enzyme had minimal activity of 44.28 ± 3 U/mg on wheat starch and 12.65 ± 0.85 U/mg on potato starch. In agreement with the results in this study, amylase cocktail produced by *A. niger* exhibited superior degradation of raw starch from wheat flour compared to SAN Super 240 L, a widely employed industrial amylase cocktail [64]. The crude enzyme from *A. oryzae* LS1 hydrolyzed soluble starch, corn starch, dextrin, and potato starch [65]. The crude amylase from *Talaromyces islandicus* AUMC 11,391 digested raw starch from maize, sorghum, wheat, rice, and oats, as well as soluble starch [46]. Soluble starch and cassava starch were hydrolyzed by 85% and 70%, respectively, by the crude enzyme generated by *Streptomyces erumpens* MTCC 7317 [66]. The amylase derived from *Bacillus licheniformis* AT70 exhibited significant hydrolytic activity on raw corn starch [67].

The influence of metal ions and inhibitors on our purified amylase revealed patterns that are broadly consistent with, but also distinct from, previous reports on fungal amylases. Among the tested additives, Mn²⁺ (as MnSO₄) and Zn²⁺ (as ZnSO₄) exhibited the strongest enhancement, increasing residual activity to 155.3% and 151.4%, respectively. This strong activation by Mn²⁺ aligns with previous findings for several fungal amylases, including those from *Penicillium citrinum* HBF62 [68], *Preussia minima* [69], and *Penicillium oxalicum* [70], suggesting that Mn²⁺ may play a conserved structural role in stabilizing the active conformation of *Penicillium* amylases, potentially through coordination with acidic amino acid residues in the catalytic domain. The pronounced activation by Zn²⁺ (151.4% residual activity) is more unusual, as Zn²⁺ has been reported to inhibit or have no effect on many fungal amylases. For instance, Zn²⁺ inhibited the amylase activity of *Aspergillus niger* JGI 24 [71] and showed no significant effect on *Scytalidium thermophilum* amylase [72]. The strong enhancement observed in our study may reflect a unique structural feature of the *P. goetzii* amylase, such as an atypical metal-binding site that accommodates Zn²⁺ as an activator rather than an inhibitor. This distinct property could be exploited in industrial applications where Zn²⁺-containing formulations are used, though further structural studies (e.g., X-ray crystallography or site-directed mutagenesis) would be required to confirm the molecular basis of this activation.

Other divalent cations, including Co²⁺ (139.2% residual activity), Mg²⁺ (129.2%), Fe²⁺ (125.6%), and Ca²⁺ (124.0%), also enhanced enzyme activity to varying degrees, consistent with the general pattern that divalent cations often serve as cofactors for fungal amylases, likely by stabilizing the enzyme-substrate complex or participating in catalytic water coordination [60]. Notably, Ca²⁺—which is essential for the structural integrity of many bacterial and fungal amylases—showed moderate activation (124.0%) rather than absolute dependence, suggesting that our enzyme retains significant activity without exogenous Ca²⁺, a feature that may be advantageous in Ca²⁺-limited industrial processes.

As expected, the anionic detergent SDS strongly inhibited enzyme activity (58.8% residual activity), indicating that the enzyme requires intact tertiary structure for catalysis and is susceptible to denaturation by amphipathic molecules. EDTA also reduced activity (77.5% residual activity), confirming that metal ions are important for full catalytic function, although the incomplete inhibition suggests that the enzyme does not have an absolute requirement for exogenous metal ions, consistent with the moderate activation observed for Ca²⁺ and other divalent cations. In contrast, Ni²⁺ was inhibitory (73.4% residual activity), a finding consistent with previous reports on Mucor sp. amylase [73] and other fungal enzymes, where Ni²⁺ likely displaces essential metal ions from the active site or induces unfavorable conformational changes. Overall, the metal ion response profile of our cold-adapted amylase from *P. goetzii* shares many features with mesophilic fungal amylases (particularly Mn²⁺ activation and SDS/EDTA sensitivity) but also exhibits distinctive properties (notably Zn²⁺ activation) that may reflect adaptations to cold environments and warrant further investigation. Furthermore, Araujo et al. [57] employed Kohonen Self-Organizing Maps (KSOM), an artificial neural network, to identify non-linear patterns in solvent-enzyme interactions, revealing that aprotic polar solvents (acetonitrile, DMSO, DMF) activated cellulolytic enzymes while protic solvents (methanol, ethanol) caused inhibition. This approach could be valuable for future studies investigating the stability of our cold-adapted amylase in the presence of organic solvents or detergent formulations. Notably, contrasting metal ion effects were observed between the two studies: while Araujo reported inhibition by Zn²⁺ and activation by EDTA for their cellulases, our cold-adapted amylase was strongly activated by Zn²⁺ (151.4% residual activity) and inhibited by EDTA (77.5% residual activity). These opposing findings highlight the enzyme-specific nature of metal ion interactions and underscore the unique metal-binding characteristics of our cold-adapted amylase, which may be related to its structural adaptations for low-temperature catalysis.

Kinetic constants for the purified amylase were determined for six starch types: soluble starch, sorghum starch, oat starch, corn starch, wheat starch, and rice starch. The corresponding K_m_ values were 575.75, 59.1, 149.27, 195.9, 81.44, and 37.48 mM, respectively, while V_max_ values were 1250, 136.98, 108.7, 156.25, 5, and 46.5 µmol/min, respectively. Numerous studies have determined K_m_ and V_max_ values for fungal amylases. In this regard, K_m_ and V_max_ values for amylase from *Trichoderma harzianum* in the hydrolysis of potato soluble starch and glycogen were 6.53 mg/mL and 4.5 mg/mL, and 2 µmol/min and 2.2 µmol/min, respectively [74]. Employing soluble starch as the substrate, α-amylase derived from *Aspergillus penicillioides* demonstrated Km and Vmax values of 5.41 mg/mL and 1.05 µmol/min, respectively [75]. Purified α-amylase from *Penicillium camemberti* PL21 demonstrated a K_m_ value of 0.92 mg/mL for soluble starch [61]. The K_m_ and V_max_ for amylase of *Talaromyces islandicus* AUMC 11931 were 132.1 mg/mL and 60.6 µmol/min, respectively [46].

## Conclusions

This study successfully demonstrates an integrated valorization strategy in which potato peel waste—an abundant agro-industrial by-product—serves as a low-cost, renewable substrate for producing a cold-adapted amylase from *P. goetzii* AUMC 498 under SSF. The purified enzyme exhibits several features that position it as a promising biocatalyst for energy-efficient industrial processes: (i) optimal activity at 25 °C and pH 5, well-suited for low-temperature applications; (ii) strong preference for sorghum starch (594.7 U/mg), indicating substrate specificity that could be exploited in specialty starch processing; (iii) marked enhancement by Mn²⁺ and Zn²⁺ (up to 155% residual activity), offering opportunities for activity modulation in formulated products; and (iv) broad substrate specificity across six starch types, reflecting catalytic versatility. From an applied perspective, the ability of this amylase to function efficiently at ambient temperatures (25 °C) while retaining measurable activity at 10 °C (the production temperature) makes it particularly attractive for industries seeking to reduce energy consumption and thermal damage to heat-sensitive substrates. Potential applications include cold-water detergents, textile desizing, and starch processing. Furthermore, the use of potato peel waste as a fermentation substrate directly addresses two pressing challenges: the environmental burden of agro-industrial waste disposal and the high cost of enzyme production. By converting a disposal problem into a value-added resource, this work provides a scalable and economically viable model for green biotechnological manufacturing. In conclusion, this study not only introduces a novel cold-adapted amylase from a previously unexplored *Penicillium* species but also establishes a sustainable production platform that aligns with circular economy principles. Future research should focus on process scale-up, enzyme immobilization for reusability, and molecular engineering to further enhance catalytic efficiency and thermal stability at low temperatures.

## Data Availability

“The dataset generated and/or analyzed during the current study is available in the GenBank: Penicillium goetzii strain AUMC 498 internal transcribed spacer 1, par—Nucleotide—NCBI; accession number PQ637268”.
